# A Plea for Thorough Dental Education

**Published:** 1904-08

**Authors:** Leo Green


### A PLEA FOR THOROUGH DENTAL EDUCATION.[1]

BY LEO GREEN, A.B., D.M.D.

This paper presents at its inception a rather discouraging aspect. The subject is so broad that it is difficult to limit it to a short paper, and yet the omission of a single phase would seem to disturb the sequence of ideas. I expect to bring up some questions that have caused no little difference of opinion in the past, but yet I want to make it clear that I am not bringing an indictment against any one institution or individual, but am merely trying to indicate the faults in the general system of dental education as I see them.

The first part of my paper will deal with the preliminary education of the student and his preparation for entrance into the professional school. I will then pass to the instruction in the dental schools and examine the methods there in vogue, both as to the theoretical and clinical training. We will then discuss the examinations for State licenses, and some professional ideals (or want of them).

But to return to the preliminary education of the dental student, and by that I refer to his mental and manual training prior to his entrance into the professional school. I regret to say that the present method of preparation for our professional career is entirely inadequate, and that the average dental student has no better education than the average clerk. In fact, a large proportion of our dental students have been unsuccessful in a mercantile career and start in, quite late in life, to study dentistry as a last resort. Hurried preparation in a cramming school, or with a tutor, enables them to pass the entrance examinations for the dental schools, or to satisfy the requirements of the State Board of Regents. Or, on the other hand, we find our material in the short course students of the high schools.

Since I wrote this paper, it was my good fortune to be presented with a copy of an article written by Dr. Harry Carlton, dean of the Dental Department of the University of California. I quote from his article as follows:

---

[1] Read before The New York Institute of Stomatology, April 5, 1904.

"How few professional schools have entrance requirements that amount to much; they may have requirements that look well in the catalogue or annual announcement, but most accept without question or investigation certificates from teachers or obscure preparatory schools, and the like. There are approximately sixty dental schools in America. To quote from a recent compilation, three have no express admission requirements; eighteen have purely grammar-school subjects for entrance; eighteen more, first year standing in high-school work; eleven have requirements covered by two years of high-school work; and six require a three years' high-school course; none of them, as much as is required for admission to any reputable college."

A man undertaking the study of any profession with such a foundation is very much handicapped, unless he is an exceptionally capable student. No officer enters the united service with so little training as our own students exhibit, unless he is on very friendly terms with the administration. No engineer, or lawyer, or teacher prepares for his life's work by the short route. We cannot erect a building on a soft foundation, and cannot turn out an educated, liberal-minded man, be he lawyer or dentist, with a professional education imposed upon mental unfitness. For our average student the study of anatomy, physiology, chemistry, and bacteriology presents a difficult and disagreeable problem, and a lack of respect for the laws of hygiene and pathology remains with him in later years of practice.

The higher classes of professional schools are beginning to demand a college degree as a requisite for admission, and we must soon fall in line. The advantages of this standard must be evident. At present our schools require only what the law demands, and in the case of foreigners or man intending to practice abroad, many concessions are made. A man is prepared, in a measure, to study any profession when he has received a college education. He is all the more prepared to study any one profession when his education has to some extent been shaped to that end in view. If his fingers as well as his mind have been trained, then has the prospective dental student received an almost ideal preparation. When a student enters Columbia University, for instance, with the ultimate intention of studying medicine, he takes the prescribed college courses and chooses such elective subjective as will be helpful to him in his medical course. At the end of three years, he is ready to enter the medical school, with a good education, ability to study and grasp new facts, and, if he has chosen his electives wisely, with a fund of information that will lead up to his medical training. The future dental student could likewise take up such elective courses as would assist him in his dental studies, and, furthermore, apply himself faithfully to manual training, to educate his fingers. Dr. Maurice Richardson, the famous Boston surgeon, attributes his skill to early training at the forge; and I have frequently heard surgeons attribute their dexterity to training in wood work, carving, painting, etching,—in short, to various forms of manual training.

You will say that this long college training entails too heavy a burden and expense upon the poor student. For the benefit of the multitude, the individual must always suffer; and yet, with the many scholarships and opportunities for self-support that our colleges offer, the ambitious student is rarely barred from an education because of his lack of means. For a simple dental operation no great learning or manual skill is necessary, but to keep in touch with the progress of dental knowledge, and to maintain dignified relations with the medical profession, a higher mental standard for our students is imperative. The lack of courtesy, or recognition (so called), from the medical profession of which dentists so frequently complain is due largely to a lack of confidence. Laymen as well as professional men are wont to look upon us as mere skilled mechanics. It is only within the past few years that the government has added dentists to the military service; and the rank of these contract dental surgeons, as compared with the collateral branches of the service,—the medical, the legal, and the clerical,—is not one to be proud of. We have been slow in making the public see the importance of our services, because we have been slow in educating ourselves. The greatest enemies to our professional progress are those men whose slogan is, "Too much theory and too many lectures." If their theory is correct, our schools are unnecessary; an apprenticeship in the office and mechanical laboratory would suffice. We must sooner or later realize that so long as our educational standards are low, so long shall we fail to attract a superior class of men to our ranks. Dr. Carlton, in referring to a paper written by Dr. Kirk and printed in the *Dental Cosmos*, says,—

"He also refers to the demand now being vigorously made from many quarters, that dentistry be classed as an 'independent' profession and given equal status in the specialties of the healing arts. I quite agree with him that much, aye, very much, remains to be done before that recognition, as such, can be accorded. If we would have the respect and regard of other professions we must first respect ourselves. Dentistry should be a full profession and recognized as such, but it only will be when the character of the men who comprise it bring its recognition up to what it should be. The conferring of the dignified degree of Doctor upon men of deficient culture is wrong, and for this reason we fail of recognition not only abroad, but at home. I have said that any increase in dignity and professional status must come from within the great body of the profession itself. To gain these ideals and make the profession what it should be a high grade of young men must be attracted to its ranks. As soon as the best men are attracted, excellent work will really be done, and the development will then be a natural one."

The announcement of a four years' dental course looks imposing in print, but does it really indicate an *advance* in dental education? Until recently, a "year" in most dental schools consisted of five or six months; and terms of more than seven months are still rare. The course of instruction and corps of instructors as represented by many schools in their annual announcements are a delight to the eye; but how different is the reality. Some few schools there are whose faculties plan out a nine months' course, and faithfully carry out this schedule. But is it not natural that students will flock to those institutions making the least demands of them in the way of time and effort? The history of our dental schools during the past twenty-five years has had its full measure of scandal. The disclosure of fraud and the sale of diplomas have incurred for us, from time to time, the distrust of foreign authorities. Less than ten years ago the State authorities were called upon to admonish the officials of one of our local institutions for neglect,—a neglect which nearly resulted disastrously for the interests of the students. Of course, exposure and official prodding have accomplished much in the way of reform, but there is still much to be desired.

An unwise feature in some of the courses is the unnecessary repetition of lectures. Men are called upon to listen to the same sets of lectures several years in succession. A lack of interest and a tendency to cut hours are the inevitable results. Such courses seem to be designed to prepare men up to the hour to pass examinations, which is not the ultimate end of education; it should be merely a feature. And yet the lectures in operative and mechanical dentistry are far superior to the clinical training in these departments. The demonstrators are for the most part inexperienced, recent graduates, for whom the ridiculously small salary is some inducement.

Furthermore, the students are wont to look upon the lecture course as a necessary evil, and as independent of and unessential to the clinical training. If this is not a fault of the system of education, at whose doors shall we lay the blame? The requirements in the actual practice of operative and mechanical dentistry are for the most part inadequate. There are other faults of the system of education to which I could call attention, but time and space prevent me from going into details. I must, however, take advantage of this opportunity to call attention to the questionable practice of permitting students to substitute apprenticeship instruction in operative and mechanical dentistry for the regular college courses in these departments.

Now, as to the annual examinations; the number of failures in the last year is so large, as compared with those of the previous years, that we naturally look for an explanation. The trouble seems to be that the examinations of the first two years are conducted in too loose a manner, and that the weeding-out process occurs only at the eleventh hour. College authorities should indicate to the impossible student, at the earliest opportunity, that his sphere of usefulness lies elsewhere.

Under the present condition of varied efficiency of our dental schools the addition of a fourth year will not produce a new era in dental education. We see in the rulings of the National Association of Dental Faculties the same error that underlies the workings of the labor unions. If all men were of equal ability, the labor union system would be fair; but it is manifestly unjust to be obliged to pay the poor workman at the same rates as the skilled, or to require the sluggard to work only as many hours as his more diligent brother and for the same hire. As it is with men, so it is with colleges. The National Association classifies the thorough institution with the lax one, and lays down a general rule for them all. It is fair to presume that you can learn more about dentistry in nine months than you can in seven; and you must expect better results from an institution which maintains a high standard equally to all, than from one which lets down the bars so that the lame sheep can pass through. Besides, the entrance requirements of some institutions produce a body of students capable of learning and thoroughly assimilating more in one year than those of another institution in two years. I am not opposed to a four years' dental course; under certain conditions I believe such a course would be admirable, and I will attempt to outline these conditions. Start with students *properly qualified to study a profession.* Insist upon their preliminary education being of a high standard and in a measure preparatory to their prospective training; this will enable the college faculties to eliminate such courses as chemistry and physics, which now form part of the dental curriculum. At the end of three years examinations for the degree may be held and successful applicants rewarded accordingly. For the fourth year I would outline a course of clinical work. You may ask, What will be gained by dividing the course in this way? After the student has received his degree, he is relieved of the worry of examinations and attendance at lectures, which, in a measure, must conflict with his clinical work. His opportunities for observation will be broader than before, and unhampered; and any taste for original research can be fostered. The results altogether will be for the good of the public, as well as of the individual.

One of the features peculiar to our form of government is the sovereign right of the State to make laws for its own government. While this has many advantages, it likewise has its disadvantages. A notable instance of the latter is the conflict of the divorce laws, which has brought both trouble and ridicule. The lack of uniformity of the laws regulating the practice of medicine and dentistry in the various States is also to be regretted. This difficulty could be remedied by a national standard of examination, which should be no less exacting in its requirements than the most rigid tests now in force. The English system, whereby a licentiate under the home law is permitted to practise in the colonies, seems eminently fair and is certainly consistent.

Too many of our practitioners suffer from a want of self confidence and individuality, which in itself is a confession of poor training. Men are too apt to accept, unquestioned, the results that others offer; new drugs or patent preparations are readily accepted as a panacea for all ills. Moreover, we have been afraid in the past, to cry out against the commercial tendency in our ranks. We have failed to distinguish the men who labor for the benefit of the profession above those who work only for their personal profit through the dental supply houses. We are either too indifferent to this condition, or else too many of us are involved in these commercial transactions. It is difficult to make many of our men understand that, while they may have a legal right, they have no moral right to conceal from the profession any useful ideas expressed in the form of new drugs, formulas, instruments, or methods of operation, or to market out the products of these ideas after having secured them by patents. Do these men ever stop to consider how slow would have been the advance of our professional knowledge, and how little they would know to-day, if all progressive thought had been veiled in secrecy or inventive genius had invested its products with patents?

The worst feature of the commercial tendency—and the most degrading—is the rapid growth of the advertising dental companies. A representative of one of our local newspapers recently asked me what form of legislation could be invoked to drive these parasite growths out of existence. Legislation alone could take care of present conditions, but the bright hope for the future lies in education. Education will take care of this and other degrading tendencies in our profession,—that I have already indicated. As regards legal enactments, I would like to quote the English law. Every candidate on being admitted as a licentiate subscribes to the following declaration:

"I do solemnly and sincerely declare that I will exercise the several parts of my profession, to the best of my knowledge and ability, for the good, safety, and welfare of all persons committing themselves or committed to my care; and I hereby promise as a Licentiate in Dental Surgery, that I will not advertise or employ any other unprofessional modes of attracting business, nor will I allow my name to be connected with any one who does so; and that I will loyally obey all by-laws of the Faculty made or to be made for the Licentiates in Dental Surgery."

"Any Licentiate who may be proved, to the satisfaction of the Council, to have violated the obligations in the foregoing declaration shall, if the Faculty so decide, render himself liable to the forfeiture of his diploma and to his name being erased from the list of Dental Licentiates."

Some such legislation might be enacted to advantage in this country.

In closing I wish to pay tribute of my respect and admiration to those institutions which have stood for higher dental education, and to those men of the present and past generations who have devoted their best efforts for the benefit of their profession and mankind.

